# Antimicrobial resistance patterns, clinical features, and risk factors for septic shock and death of nosocomial *E coli* bacteremia in adult patients with hematological disease: A monocenter retrospective study in China

**DOI:** 10.1097/MD.0000000000006959

**Published:** 2017-05-26

**Authors:** Jie Ma, Ning Li, Yajie Liu, Chong Wang, Xiaoyan Liu, Shengmei Chen, Xinsheng Xie, Silin Gan, Meng Wang, Weijie Cao, Fang Wang, Yanfan Liu, Dingming Wan, Ling Sun, Hui Sun

**Affiliations:** Department of Hematology, The First Affiliated Hospital of Zhengzhou University, Zhengzhou, China.

**Keywords:** antimicrobial resistance patterns, clinical features, death, *E coli* bacteremia, hematological center, risk factors, septic shock

## Abstract

The aim of this retrospective analysis was to evaluate the antimicrobial resistance, clinical features, and risk factors for septic shock and death of nosocomial *E coli* bacteremia in adult patients in a single hematological center in China. A retrospective case-control study of 157 adult hematological patients with 168 episodes of *E coli* bacteremia was initiated from April 2012 to July 2015. Antimicrobial susceptibility as well as antimicrobial co-resistance rates were analyzed. Clinical features and outcomes were also studied. In addition, risk factors for septic shock and death were investigated. Among the 553 positive blood isolates during the study period, the prevalence of *E coli* was 33.3% and ESBL production strains represented 61.9% of those examined. In all the *E coli* strains isolated, 85.6% were multidrug-resistance (MDR), 2.4% were extensive drug resistance (XDR), and 6.0% were resistant to carbapenems. More MDR phenotype was noted in ESBL-EC strains (98.6% vs 62.8%, *P*<.001) and isolates from neutropenic patients (98.6% vs 62.8%, *P* < .001). In the antimicrobial susceptibility test, carbapenems and amikacin exhibited not only higher in vitro activity against *E coli* (94.0% and 92.0%, respectively), but lower co-resistance rates to other antibiotics. Carbapenem resistant strains retained full sensitivity to tigecycline and 60% to amikacin. Piperacillin/tazobatam was the third sensitive drug to both ESBL-EC (77.1%) and non-ESBL-EC (86.0%). In our series, 81.6% episodes received appropriate initial antibiotic treatment and no significant decrease in it was found in bacteremia due to ESBL *E coli* and patients with neutropenia, septic shock. Septic shock was noted in 15.5% patients and the overall 30-day mortality rate was 21.7%. Multivariate analysis revealed that induction chemotherapy (OR 2.126; 95% CI 1.624–11.332; *P* = .003) and polymicrobial infection (OR 3.628; 95% CI 1.065–21.219; *P* = .041) were risk factors for septic shock, whereas male (OR 2.223; 95% CI 1.132–12.022; *P* < .01) and septic shock (OR 52.359; 95% CI 19.951–292.690; *P* = .030) were risk factors for death.

In the hematology department, ESBL-producing and MDR are widely prevalent in *E coli* bacteremia which is still a major life-threatening problem, especially for patients with septic shock. For empirical antimicrobial therapy, combination based on aminoglycoside, especially amikacin, will be helpful to increase the antimicrobial coverage against ESBL-EC while combining tigecycline with aminoglycoside should be considered for seriously carbapenem-resistant infectious patients.

## 1 Introduction

Over the last decade, advances in treatment approaches have improved the prognosis of patients with hematological disorders, especially hematological neoplasms. Unfortunately, bloodstream infections (BSIs), one of the most common severe infections in hematology department, is still the important complication contributing significantly to extended hospitalization and increased mortality. Recently, a shift towards Gram-negative bacteria has been noted in the bacterial epidemiology of hematological patients.^[1–3]^ Moreover, the production of extended-spectrum β-lactamases (ESBLs) has drawn great attention due to the spread of antimicrobial resistance. *Escherichia coli* (*E coli*) has become an important ESBLs producer since 2000,^[4–6]^ and a significant increase in the number of ESBLs producing *E coli* (ESBL-EC) BSIs has been reported by several studies.^[7–10]^ Meanwhile, ESBLs production adversely affected the outcomes of *E coli* bacteremia in cancer, especially patients with a hematologic malignancy.^[11]^ ESBL-producing *E coli* bacteremia among patients with hematological malignancies has been reported. However, risk factors for mortality, drug resistance, and impact of antimicrobial therapy on outcome among different studies still remain controversial.^[8–10,12–14]^ The discrepancy may be related to the local prevalence of pathogens causing infection and their antibiotic susceptibility, which may change over time. Currently, although some results of antimicrobial resistance of *E coli* in certain hematologic disorders were reported, information about antimicrobial resistance, especially antimicrobial co-resistance of *E coli* causing bacteremia in adult patients in the whole hematological center was limited. Furthermore, the distinctive clinical characteristics of hematological patients with *E coli* bacteremia have not been well established. Therefore, in this study, we collected clinical data of hematological patients with *E coli* bacteremia, analyzed the antimicrobial sensitivity as well as antimicrobial co-resistance rates of *E coli*, compared the clinical characteristics and investigated the risk factors for septic shock and death of *E coli* bacteremia patients, in order to guide the clinical recognition, and implement effective treatment decisions.

## 2 Materials and methods

### 2.1 Study design and patients

Approved by the ethics committee of the first affiliated hospital of Zhengzhou University, Henan Province, China, we conducted a retrospective observational study at our hospital. From April 1st 2012 to July 31st 2015, hematological patients aged ≥14 years were enrolled if they had at least 1 episode of *E coli* bacteremia. Clinical data were collected from medical records and no additional medical procedures were performed. We analyzed the characteristics of patients with *E coli* bacteremia from the following aspects: the presence of ESBL, neutropenia, septic shock, adequate initial antimicrobial therapy, and antimicrobial susceptibility.

The following data were collected: age, gender, underlying diseases, comorbidities, absolute neutrophil count, presence of septic shock, chemotherapy treatment (30 days prior to the index infection), receipt of glucocorticoid or immunosuppression agents within 30 days prior to bacteremia, antimicrobial susceptibility profile, antimicrobial agents applied during the previous 30 days, and the presence of a PICC. Clinical outcome (30 days after the infection episode) was classified as alive, death, or lost to follow-up.

### 2.2 Bacteriology and antimicrobial susceptibility testing

Identification of *E coli* strains and susceptibility testing were performed using standard microbiologic methods with an automated system in the microbiology laboratory. Antimicrobial susceptibility testing was performed using the Kirby–Bauer disk diffusion method. Production of ESBLs was confirmed using the double-disk synergy test in accordance with the Clinical and Laboratory Standards Institute standards.^[15]^

### 2.3 Definitions

The date of collection of the blood culture which yielded *E coli* was regarded as the date of bacteremia onset. A relapsing bacteremia was considered as a second episode of bacteremia caused by *E coli* during the study period. Polymicrobial bacteremia was defined as isolation of *E coli* and an additional bacterium from the blood at the time of the diagnostic blood culture. Nosocomial infection was defined as an infection that occurred >48 hours after hospital admission, an infection that occurred <48 hours after admission to the hospital in patients that had been hospitalized in the 2 weeks prior to admission, and an infection that occurred <48 hours after admission to the hospital in patients that had been transferred from another hospital or nursing home.^[16]^ Neutropenia was defined as an absolute neutrophil count (ANC) <0.5 × 10^9^/L. Septic shock was defined as sepsis associated with evidence of organ hypoperfusion and a systolic blood pressure <90 or >30 mm Hg less than the baseline or a requirement for the use of a vasopressor to maintain blood pressure. Antibiotic exposure was defined as any antibiotic therapy >24 hours but <30 days prior to the time when the positive blood cultures were drawn. Adequate initial antimicrobial therapy was defined as at least 1 antibiotic agent administrated by the intravenous route within the initial 24 hours of index blood drawn and should be active in vitro against the infecting microorganism.^[17]^ Glucocorticoid/immunosuppressive therapy was identified as receiving equivalent to ≥20 mg prednisone/day for at least 1 week or receiving cyclosporine, antithymocyte globulin (ATG), and tacrolimus within 30 days before onset of bacteremia. Day 30 mortality was defined as the time from the positive blood culture until death. All antimicrobial susceptibility results that fell into the intermediate category were presumed to be resistant in this study. According to the guidelines recommended by joint initiative of the European Center for Disease Prevention and Control (ECDC) and the centers for Disease control and Prevention (CDC),^[18]^ the isolates showing non-susceptibility to at least 1 agent in 3 or more antimicrobial categories were identified as MDR, non-susceptibility to at least 1 agent in all but 2 or fewer antimicrobial categories were identified as XDR and non-susceptibility to all agents in all antimicrobial categories were identified as PDR.

### 2.4 Statistical analysis

Student's *t* test was used to compare continuous variables, and Pearson chi-square test or Fisher^'^s exact test was used to compare categorical variables and percentage. Odds ratios (ORs) and 95% confidence intervals (CIs) were calculated to determine the strength of associations that emerged. *P* values ≤.05 were considered statistically significant, and all probabilities were 2-tailed. Variables that were associated with septic shock and death in the univariate analysis (*P* < .05) were entered into a multivariate logistic regression analysis using stepwise selection. SPSS (version 22.0) was used for all analyses.

## 3 Result

### 3.1 Patient information, incidence, and cause of *E coli* BSI

A total of 5223 samples from the cases with suspected bacteremia were analyzed in our study. In total, 553 microorganisms were isolated and *E coli* was the most common species, accounting for 184 episodes (33.3%). A total of 168 episodes of *E coli* bacteremia occurred in 157 patients were analyzed, including 8 (5.1%) patients with 2 episodes of bacteremia and 1 (0.6%) with 4 episodes. Sixteen episodes were excluded because of non-hematological diseases. The median age of the final cohort was 39 years old with an equal gender distribution (51% female). Among the 157 patients, 77 patients (49.0%) had acute myelogenous leukemia (AML), 43 (27.4%) had acute lymphocytic leukemia (ALL), 12 (7.6%) had aplastic anemia (AA), 8 (5.1%) had multiple myeloma (MM), 6 (3.8%) had myelodysplastic syndrome (MDS), and 11 (7%) suffered from other hematological diseases. The recurrent bacteremia was observed in 9 patients (5.7%) with acute leukemia (AL), including 7 with AML and 2 with ALL. Among the total of 168 episodes, 137 (81.5%) were associated with chemotherapy, 7 (4.2%) occurred after hematopoietic stem cell transplantation, 135 (80.4%) were obtained at a state of neutropenia, and 53 (31.5%) were detected in the patients with glucocorticoid/immunosuppressive agents treatment 30 days prior to bacteremia onset. Of the total 168 episodes, 89 (53.0%) episodes were complicated by pneumonia. The 30-day mortality was noted in 34 cases (21.7%). In our study, antibiotic prophylaxis was not administered to any patient.

### 3.2 ESBL and in vitro antimicrobial susceptibility analysis

Of the 168 *E coli* isolates, 113 episodes (67.3%, 2013.4–2015.7) were detected for the production of ESBLs, and ESBL-producing *E coli* accounted for 61.9% (70). Additionally, antimicrobial susceptibility profiles for 166 isolates were available. Among these isolates, 142 (85.6%) were MDR, and 4 (2.4%) were XDR. No PDR isolates were found. However, 10 (6.0%) isolates resistant to carbapenems were detected during the study period. Among the 20 *E coli* strains isolated from recurrent patients, 77.8% (14/18) were ESBL-EC, 75% (15/20) were MDR, and 10% (2/20) were XDR. Bacteremia episodes due to polymicrobial strains were found in 8 patients, including 5 with AML, 2 with ALL, and 1 with MDS-RAEB1, accompanied by *Klebiella* pneumonia in 2 cases, and *Aeromonashydrophila*, *Staphylococcus aureus*, *Pseudomonas aeruginosa*, *Stenotrophomonasmaltophilia*, *Salmonella Dublin*, and *Aermonas Veronnibiovarsobria* in 1 case, respectively.

The top 5 *E coli*-resistant antibiotics in our study were ampicillin (91.6%), sulfamethoxazole and trimethoprim (SMZ-TMP) (80.6%), ampicillin/sulbactam (79.5%), ciprofloxacin (74.1%), and levofloxacin (73.5%). Carbapenems exhibited highest in vitro activity against *E coli* strains (94.0%), followed by amikacin (92.0%) and piperacillin/tazobatam (80.6%). As shown in Table 1, compared to ESBL-EC, non ESBL-EC showed higher susceptibility to cefepime, cefazidime, cefatrixone, and aztreonam. Despite the significant differences, both ESBL-EC and non-ESBL-EC showed lower susceptibility to levofloxacin and gentamicin.

**Table 1:**
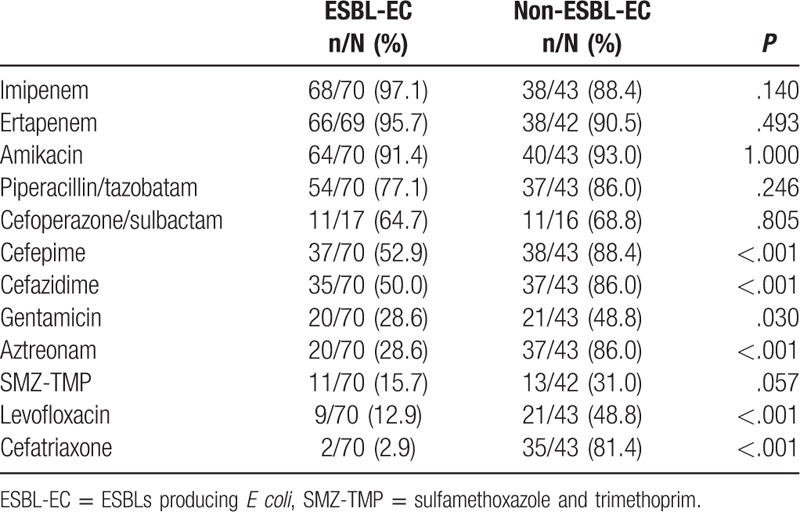
Comparison of antimicrobial susceptibility between ESBL-EC and non-ESBL-EC.

### 3.3 In vitro co-resistance pattern of antimicrobial susceptibility

Further analysis of the co-resistance pattern of antimicrobial susceptibility revealed that cephalosporins, levofloxacin, aztreonam, and SMZ-TMP had higher co-resistance rates than other antibiotics. In contrast, amikacin and imipenem exhibited lower co-resistance rates (Table 2). Strains resistant to piperacillin/tazobatam, cefoperazone/sulbactam still remained sensitivity to amikacin and imipenem. Ten (6.0%) isolates resistant to carbapenems, including 6 resistant to both imipenem and ertapenem, 1 resistant to ertapenem only, and 3 resistant to imipenem without information of ertapenem, only exhibited good sensitivity to tigecycline and polymyxin (100%, data not shown) followed by amikacin (60%).

**Table 2:**
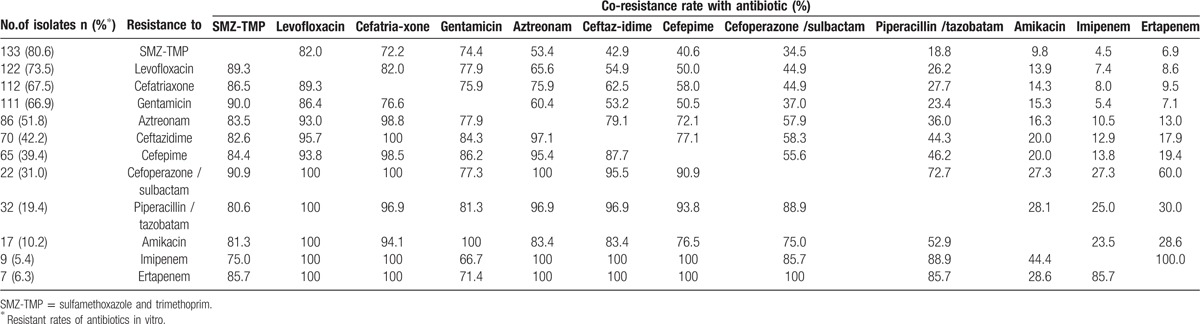
Summary of antimicrobial co-resistance rates determined for *E coli*.

### 3.4 Clinical features of *E coli* bacteremia

In the detected episodes, ESBL-producing *E coli* was the predominant species (61.9%), and the clinical features of ESBL-EC and non-ESBL-EC bacteremia were analyzed. As shown in Table 3, nearly all ESBL-ECs were MDR strains. More patients with ESBL-EC bacteremia were exposed to antimicrobial agents within 30 days prior to the onset of bacteremia (75.5% vs 46.5%, respectively; *P* = .002). Fluoroquinolone were most frequently used in the ESBL-EC group (41.4% vs 20.9%, *P* = .025), followed by carbapenem (34.3% vs 9.3%, *P* = .003). As for the appropriate initial antibiotics, no significant difference between these 2 groups was found. Although ESBL-EC was associated with higher incidence of septic shock (17.1% vs 9.3%, *P* = .246) and 30-day mortality (21.4% vs 13.9%, *P* = .321), the differences were not statistically significant.

**Table 3:**
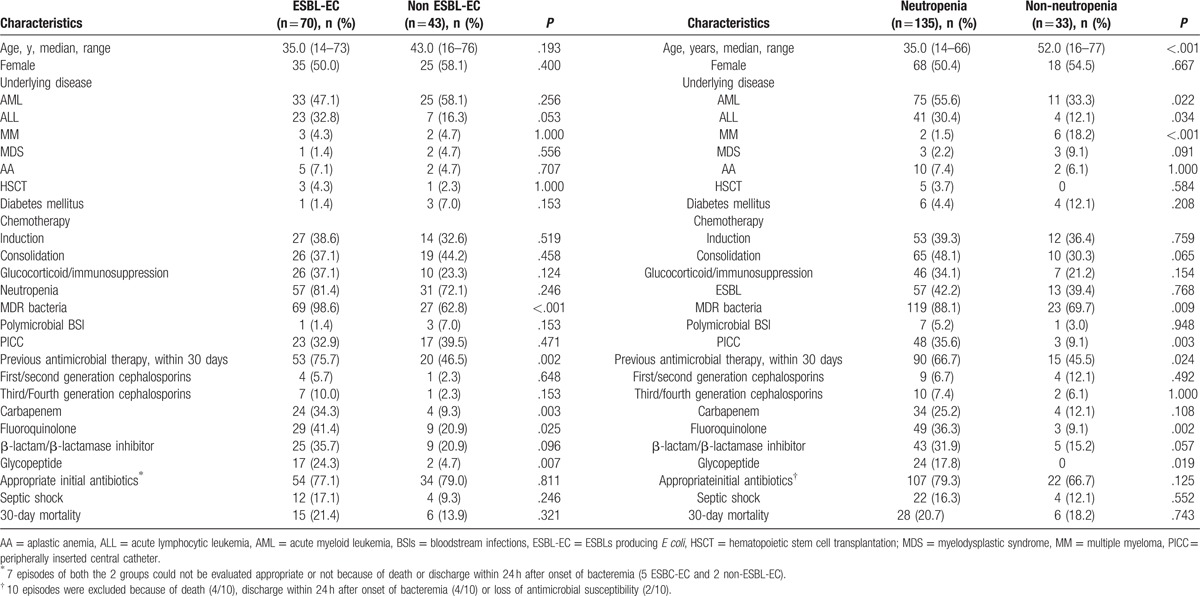
Clinical factors related to patients with ESBL-EC and neutropenia.

Clinical features of patients with neutropenia are presented in Table 3. Neutropenic patients were younger (36.3 ± 14.6 vs 50.4 ± 16.2, *P* < .001) and neutropenia was more prone to occur in AL patients. More importantly, an MDR phenotype occurred in 88.1% of neutropenic patients, which was significantly higher than that in non-neutropenic patients (69.7%, *P* = .009). More antibiotics were given to patients with neutropenia 30-day prior to the bloodstream infection onset (66.7% vs 45.5%, *P* = .024), in which fluoroquinolone were most frequently used (36.3% vs 9.1%, *P* = .002). However, the number of neutrophil was not related to the production of ESBLs, neither the risk of septic shock nor 30-day mortality.

### 3.5 Initial antibiotics treatment

Immediately after collection of the suspicious blood specimen, all patients received empirical antibiotic treatment belonging to the following classes: carbapenems (imipenem or meropenem) (67.3%), β-lactam/β-lactamase inhibitor (16.1%),fluoroquinolone (13.1%), cephalosporins (6.5%), and etimicin (4.2%). Ten episodes could not be evaluated for the initial antibiotics treatment due to death, discharge within 24 hours after onset of bacteremia or loss of antimicrobial susceptibility. Among the rest 158 episodes, 129 (81.6%) received appropriate initial antibiotics. Overall, patients treated with or without appropriated initial antibiotics had similar characteristics except that patients treated with appropriate initial antimicrobial therapy had less histories of prior antimicrobial therapy with a drug belonging to the same class prescribed empirically for the BSI (20.9% vs 44.8%, *P* = .007). Furthermore, the length of stay (LOS) (32.1 ± 23.3 vs 30.2 ± 23.9) and 30-day mortality (15.5 vs 17.2) between these 2 groups showed no statistical significances (*P* = .700 and *P* = 1.000, respectively).

### 3.6 Risk factors for septic shock and death

Risk factors for septic shock and death were illustrated in Table 4. Based on the multivariate analyses, polymicrobial infection (OR = 3.628, 95% CI = 1.065–21.219, *P* = .041) and induction chemotherapy (OR = 2.126, 95% CI = 1.624–11.332, *P* = .003) were identified as the independent risk factors for septic shock. Furthermore, septic shock (OR = 52.359, 95% CI = 19.951–292.690, *P* <.001) and male (OR = 2.223, 95% CI = 1.132–12.022, *P* = .030) had a significant association with 30-day mortality. However, no decrease in appropriate initial antibiotics treatment was noted in the patients with septic shock and death (*P* >.005).

**Table 4:**
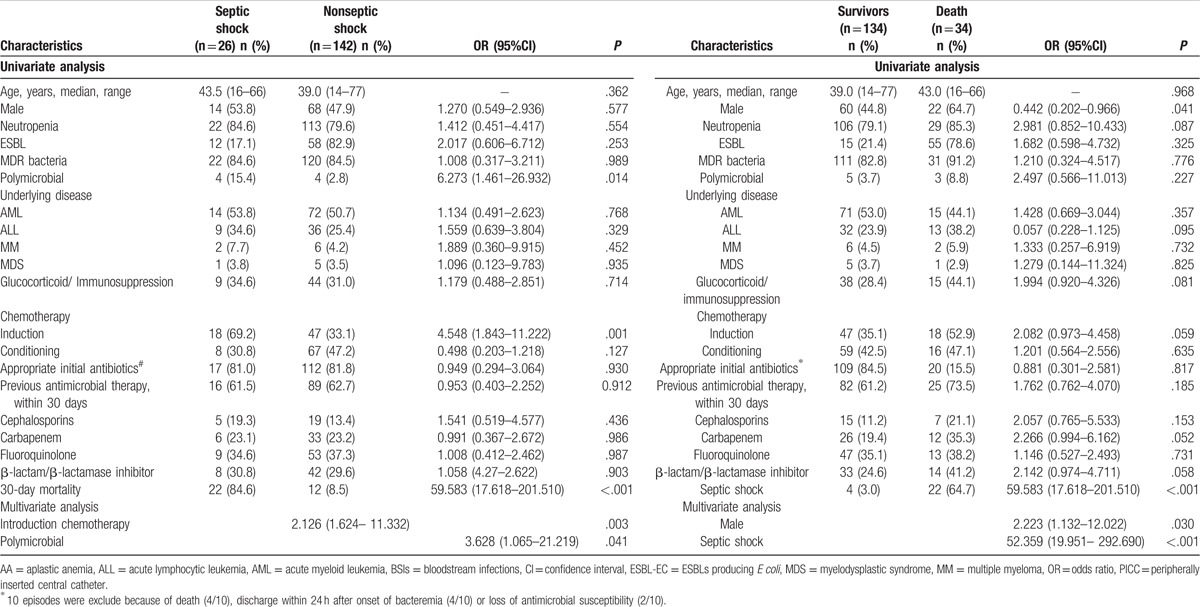
Risk factors for septic shock and death in patients with *E coli* bacteremia based on univariate analysis and multivariate analyses.

## 4 Discussion

Bloodstream infection is an important cause of morbidity and mortality in patients with hematological disorders and may contribute to delayed administration of chemotherapy and increased healthcare expenditure. *E coli* has emerged as an important pathogen in recent years.^[7,10,20]^ During the study period, most patients enrolled were immunocompromised, 85.3% with neoplastic disease, 80.3% with neutropenic and 31.5% with glucocorticoid/immunosuppressive treatment and of the overall BSIs observed in these hematological patients, *E coli* was the most common organism accounting for 33.3%. Therefore, we specifically analyzed the spectrum of susceptibility and co-resistance patterns of *E coli* isolated and we further investigated the clinical characteristics and risk factors for septic shock and death of *E coli* bacteremia episodes in 157 patients.

During recent decades, the growing involvement of ESBL-producing strains of *E coli* is one of the most significant epidemiological changes in serious infections.^[10,21–24]^ As to our study, 61.9% *E coli* tested were ESBL-producing strains. Since the ESBL genes are usually found in large plasmids which also contain other antimicrobial resistant genes, most ESBL producing organisms are MDR strains.^[25]^ The proportion of multidrug resistance *E coli* varies due to the underlying diseases and regional differences (range 31–92%).^[26–30]^ In our study, 85.6% stains isolated were MDR and 2.4% were XDR. Regarding ESBL-EC, nearly all (98.6%) were MDR. The increase in MDR *E coli* is known to be associated with hematological malignancy, prior admission to the hospital, previous chemotherapy, and antibiotic consumption.^[26]^ 85.3% patients in our study were diagnosed as hematological neoplasms and needed frequent hospitalization, chemotherapy, and antibiotic treatment, which may contribute to the high MDR rates.

Recently, a study on susceptibilities of ESBL-EC isolates causing bacteremia in South Korea reported that more than 80% of ESBL-EC were non-susceptible in vitro to ampicillin, ampicillin-sulbactam, ticarcillin-clavulanic acid, cefotaxime, and aztreonam, 32.6% and 11.7% strains were resistant to piperacillin-tazobactam and amikacin, respectively, whereas carbapenem had the lowest resistant rates (1.7–5.2%).^[31]^ We presented the similar results that carbapenems and amikacin were the 2 most effective drugs to both ESBL-EC (95.7% and 91.4%, respectively) and non-ESBL-EC (88.4% and 93.0%, respectively), followed by piperacillin/tazobatam (77.1% for ESBL-EC and 86.0% for non-ESBL-EC). Although cefepime, aztreonam, and levofloxacin remained sensitivities to non-ESBL-EC, the sensitivities to ESBL-EC were significantly lower. Carbapenems, which are generally considered as the most reliable therapeutic agents in severe infections, was the treatment choice for 67.3% episodes in our study. However, with the widespread use in clinical, resistance to carbapenems is also emerging ^[32]^ and 6% carbapenems resistant strains were found over the study period. Therefore, alternatives to the carbapenems should be considered for empirical treatment of suspected *E coli* sepsis whenever possible.^[31]^ Fortunately, comparable to carbapenems, amikacins showed the relatively high in vitro activity to *E coli* (91.4% for ESBL-EC and 93.0% for non-ESBL-EC). More importantly, in co-resistance evaluation, amikacin exhibited relatively lower co-resistance rates to cefepime, piperacillin/tazobatam, cefoperazone/sulbactam, levofloxaci, and aztreonam. Moreover, except for 100% sensitivity to tigecycline, strains resistant to imipenem remained 60% sensitivity to amikacin. These results are consistent with the previous studies,^[28,31,33]^ suggesting that amikacin would be the effective and economical choice of antibiotics for hematological patients to increase the range of antimicrobial coverage against ESBL-EC. For the infections caused by Gram-negative bacteria, antimicrobial synergy has traditionally been seen with β-lactam and aminoglycoside combination therapy. Our results of antimicrobial susceptibility and co-resistance testing indicated that the combination of aminoglycoside with piperacillin/tazobatam may be an additional alternative of empirical antibiotic therapy. This combination therapy could also be used as one of the carbapenem-saving strategies in settings with a high prevalence of ESBL-producing pathogens. But for carbapenem resistant *E coli*, combining tigecycline with an aminoglycoside will serve as the last-resort drugs for seriously infected patients.

As mentioned above, 98.6% pathogens were MDR stains in the ESBL-EC group, which is considered to be related to the high percentage of previous antimicrobial therapy (75.7% vs 46.5%, *P* = .002). Heavy antibiotic use is a risk factor for acquisition of an ESBL-producing organism.^[34]^ Several studies reported that previous use of quinolones was associated with subsequent infections caused by ESBL-producing organisms.^[35–37]^ In addition to fluoroquinolone, carbapenem was another frequently used agent for ESBL-EC. Although it has been reported in several studies that patient with an ESBL-EC bacteremia have 4 times greater overall mortality compared with non-ESBL-producing isolates,^[14,38,39]^ the effect of ESBL-EC bacteremia on mortality is still controversial.^[19,30,37]^ Some studies have shown that mortality is associated with inappropriate antimicrobial therapy, irrespective of ESBL production, whereas others have reported that the increased mortality is due to ESBL.^[19,37]^ Olson et al's^[19]^ study, in which 58.5% patients were diagnosed as hematological malignancies, showed that the presence of ESBL-EC bacteremia was not associated with day 30 mortality (30% vs 27%; *P* = . 82). Consistently, we found that although the 30-day mortality is higher in the ESBL-EC group (21.4% vs 13.9%), the difference was not statistically significant (*P* = .321). Contrary to the previous data that appropriate empirical therapy was significantly less frequent for infections caused by ESBL-producing strains,^[37]^ appropriate empirical treatment was given nearly equally in ESBL-EC and non-ESBL-EC patients (77.1% vs 79.0%; *P* = .811), which probably is the main factor that diminished the difference in mortality between ESBL and non-ESBL groups.

Hematological patients with neutropenia have the high risk for bacteremia and may present with severe sepsis and a poor outcome.^[22,40]^ 80.4% patients were neutropenic in the present study and most of them were younger with diagnosis of acute leukemia (Table 3). The neutropenia in these patients was caused by stronger chemotherapy regimen and more intensive chemotherapy courses. The percentage of MDR strains detected in neutropenic patients is significantly higher (88.1% vs 69.7%, *P* = .009) which may be related to the fact that more neutropenic patients received previous antimicrobial therapy (67.7% vs 45.5%, *P* = .024), especially fluoroquinolone (36.3% vs 9.1%, *P* = .002), a well-known risk factor for development of resistance.^[9]^ A relationship between infection with resistant bacteria and poor outcome has been reported in several settings, mainly due to a delay in the initiation of an appropriate antibiotic therapy.^[40,41]^ Our results showed a similar mortality rate in patients with or without neutropenia (20.7% vs 18.2%, *P* = .743%). The loss of negative effect of neutropenia on mortality in our study may be due to the treatment of appropriate initial antibiotics to most patients (79.3% vs 66.7%, *P* = .125), which gives survival benefit in immunocompromised patients.

Septic shock occurred in 15.5% patients in the present study. In a previous study consisting of a large proportion of young patients with hematological malignancies,^[42]^*E coli* and polymicrobial bacteremia were found to be associated with septic shock (*P* = .01). Similar to the result, our result of multivariate analysis revealed that induction chemotherapy and polymicrobial bacteremia were risk factors for septic shock in *E coli* Bacteremia. Septic shock is still a major cause of mortality in patients with hematologic diseases. Consistent with other studies,^[37,42–44]^ patients with septic shock had significantly higher 30-day mortality (84.6% vs 8.5%, *P*<.001) in spite of the fact that 81.0% patients were treated with appropriate empirical antibiotics. Further multivariate analysis showed that male and septic shock were the independent risk factors for death (OR 2.223; 95% CI 1.132–12.022; *P* <.01, OR 52.359; 95% CI 19.951– 292.690; *P* = .030, respectively). In addition, it should be noted that bacteremic *E coli* had a high diversity of genetic backgrounds and virulence factor (VF) gene profiles.^[45]^ Thus, the prognosis of *E coli* bacteremia is not only associated with host factors, but also related to pathogen features.^[45]^ Mora-Rillo et al^[45]^ found that one of the VF genes, fyuA, increased the risk of mortality, while any combination of genes encoding for P fimbriae components had a protective role. The influence of VF genes on mortality of *E coli* bacteremia in hematological patients needs further exploration.

Several limitations remain in this study. First, our analysis was retrospective and confined to a single hematology center, so the results are not necessarily applicable to other settings. Second, molecular epidemiology, for both ESBL producing and carbapenem resistant *E coli* as well as VF gene profiles of *E coli*, was not analyzed. Third, the clinical features and risk factors for polymicrobial, recurrent bacteremia, and carbapenem-resistant *E coli* bacteremia were not assessed due to the limited sample size during the study period.

In conclusion, this study shows that *E coli* is the most common pathogen responsible for bloodstream infection in hematology department. Unfortunately, most of *E coli* detected are ESBL-producing and MDR strains with emergence of XDR and carbapenem-resistant isolates, resulting in fewer treatment options and difficult empiric therapy. Based on our results, in settings with a high prevalence of ESBL-producing *E coli*, combination aminoglycoside antimicrobial therapy, especially amikacin, should be considered as an empirical therapy to reduce the risk of further development of carbapenem resistance. Combining tigecycline with an aminoglycoside will serve as the most effective drug for carbapenem resistant *E coli*. In addition, more attention should be paid to the patients with signs of septic shock, which significantly increased mortality. Thus, our findings provide significant information about the microbiological, clinical characteristics and risk factors for septic shock and death of *E coli* bacteremia, which are vital for clinical management of such kind of patients.
